# Identifying key mental health and improvement factors in hospital administrators working from home using a DEMATEL-based network analysis model

**DOI:** 10.3389/fpubh.2024.1287911

**Published:** 2024-03-19

**Authors:** Sheng Shu, Jie Zhu, Wenqing Shi, Yen-Ching Chuang, Chao Liu, Hongsheng Lu

**Affiliations:** ^1^Taizhou Central Hospital (Taizhou University Hospital), Taizhou, China; ^2^Business College, Taizhou University, Taizhou, Zhejiang, China; ^3^Institute of Public Health and Emergency Management, Taizhou University, Taizhou, Zhejiang, China; ^4^Key Laboratory of Evidence-Based Radiology of Taizhou, Linhai, Zhejiang, China; ^5^The Second Affiliated Hospital of Hebei North University, Zhangjiakou, Hebei, China; ^6^Institute for Hospital Management, Tsinghua University, Shenzhen International Graduate School (SIGS), Shenzhen, Guangdong, China

**Keywords:** mental health, work from home, Decision-making Trial and Evaluation Laboratory-based Analytic Network Process (DEMATEL-based ANP or DANP), multi-criteria decision-making (MCDM), COVID-19 normalization prevention and control

## Abstract

**Purpose:**

To identify the key mental health and improvement factors in hospital administrators working from home during COVID-19 normalization prevention and control.

**Methods:**

The survey was conducted from May to June 2023, and the practical experiences of 33 hospital administrators were collected using purposive sampling. The study examined a set of mental health factor systems. The relationship structure between the factors was constructed using the Decision-making Trial and Evaluation Laboratory (DEMATEL) method. Finally, the structure was transformed using the influence weight of each factor via the DEMATEL-based Analytic Network Process.

**Results:**

Regarding influence weight, the key mental health factors of hospital administrators are mainly “lack of coordination,” “time management issues,” and “work-life imbalances.” The influential network relation map shows that improvements can be made by addressing “improper guidelines,” “laziness due to being at home,” and “job insecurity” because they are the main sources of influence. The reliability level of the results for the network structure and weight was 98.79% (i.e., the gap was 1.12% < 5%).

**Conclusion:**

The network analysis model based on DEMATEL proposed in this study can evaluate the mental health factors of hospital administrators during the pandemic period from a multidimensional and multidirectional perspective and may help improve mental health problems and provide suggestions for hospital administrators.

## Introduction

The COVID-19 outbreak is a public health emergency of international concern that spread rapidly worldwide and gradually evolved into a pandemic with disastrous consequences (1, 2). COVID-19 seriously threatens people’s health and global security, and has caused incalculable losses to the global economy, education, and medical care (3, 4). Doctors and nurses are at the frontline of prevention and control of the COVID-19 epidemic and play a key role in preventing infection and treating patients (5). However, during the outbreak, doctors and nurses were exhausted and understaffed, posing certain risks to public health (6). The high risk of COVID-19 infection can seriously affect the mental health of doctors and nurses, and they may be anxious about infecting other personnel (7, 8). COVID-19 is one of the main representatives of sudden major infectious diseases. Hospitals are the main institutions that fight against major infectious diseases. Therefore, the related topics concerning hospitals require investigation, especially concerning major epidemics. This includes the mental health problems of anti-epidemic roles such as doctors, nurses, and administrators need special attention.

During the fight against COVID-19, some studies focused on the mental health of doctors and nurses because they were frontline workers in the fight against the epidemic. For example, a survey in a Spanish general hospital found that more than 36% of the staff were infected with COVID-19, of whom 32% were asymptomatic (9). One study conducted a psychological survey of 9,138 medical staff and found that 45.7% of them had mental disorders, of which 14.5% were even more serious (10). In addition, one study found that 80% of confirmed patients still suffered from fatigue, cognitive impairment, dyspnea, and other sequelae after recovery (11). Doctors and nurses are important actors in the fight against COVID-19. Doctors and nurses who are infected and isolated leave the clinical front line, which causes a shortage of pandemic prevention personnel and increases the workload of other colleagues (12). Simultaneously, they worry they will infect their families, relatives, and neighbors (13). The above indications show that a shortage of personnel, self-isolation, illness, and death of confirmed patients all cause an emotional burden on doctors and nurses (14). With the epidemic changing from confrontation transformation to normal prevention and control, office and study environments have shifted online. Therefore, people who work or study at home also merit attention.

During the epidemic, to reduce cross-infection in hospitals and reduce the ability to prevent and control the epidemic, hospitals advocate for non-major medical or nursing posts to work at home, among which administrative staff are the main group working at home (15). According to one survey, most administrators who work from home, such as medical staff, also experience mental health problems (16). However, hospital administrators, as the main employees working at home, engage in many complicated and tedious administrative tasks. Their mental health has also been seriously affected; however, little attention has been paid to this issue. Therefore, it is necessary to study mental health problems faced by hospital administrators working at home (17). To address this research gap, it is necessary to analyze the key factors of hospital administrators’ mental health to serve as a reference for the mental health management of hospital administrators during the potential major infectious epidemic in the future.

Mental health is usually evaluated from multiple factors/dimensions, which are suitable for multi-criteria decision-making (MCDM) as an analytical method. Moreover, the Decision-making Trial and Evaluation Laboratory-based Analytic Network Process (DEMATEL-based DANP or DANP) method can establish an influence relationship structure diagram and assess influence weights. The influence relationship structure diagram can help decision-makers understand the interaction between all factors (18). The influence weights can help decision-makers identify key factors in the system (19).

## Materials and methods

### Research design and analysis process

**Figure 1:**
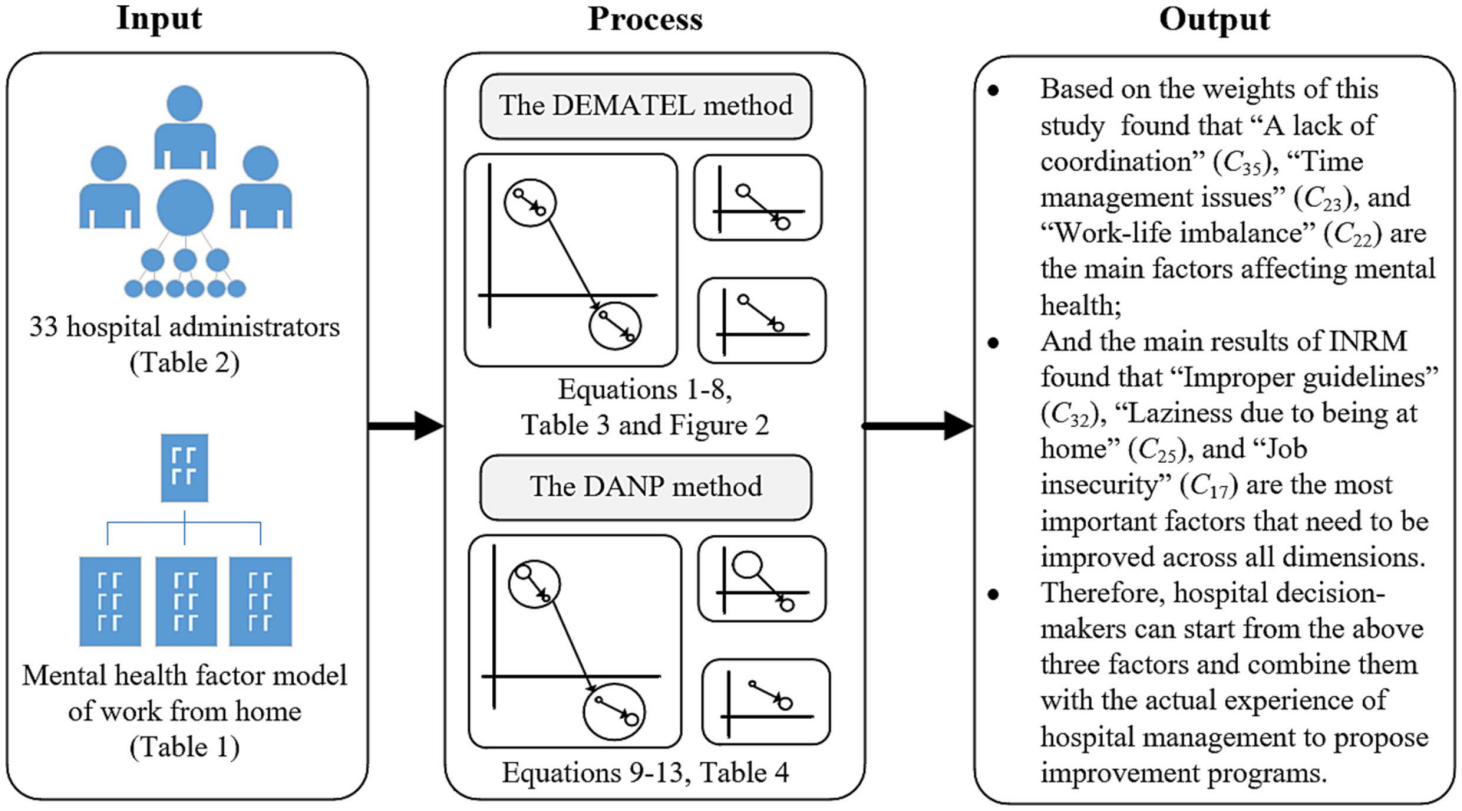
The research design flow chart.

To understand the impact of homework on the mental health of hospital administrators during a major epidemic, this study quantitatively transforms the practical experience of hospital administrators into numerical values. It visualizes the relationship structure and corresponding influence weights of their psychological factors. Hospital policymakers can distinguish the degree of interaction of psychological factors through quantitative numerical values and identify their priorities. In this study, the design and process are divided into three stages: Stage one is designing the questionnaire based on the DANP method and the model of mental health factors. In stage two, the questionnaire collects the practical experience of 33 hospital administrators by purposeful sampling method. Then, the degree of interaction of mental health factors is calculated by the DEMATEL method, and an influential network relationship map (INRM) is constructed. In stage three, the total influence matrix produced by the DEMATEL method is converted into a weight. The survey window was from May to June 2023, and the research design flow chart is shown in Figure 1.

### Mental health factor model of work from home

Memon et al. (20) used the qualitative phenomenological design method to explore home office employees’ life experiences during COVID-19 and recruited 41 employees using snowball sampling. The study (20) followed the thematic analysis steps defined by Braun and Clarke (21) to form a mental health factor model for home offices. In this study, we analyzed five themes surrounding working from home, namely technical problems, work-related stress, non-work stress, communication problems, and motivation and productivity problems. Because this study focuses on hospital administrators, technical issues, we excluded motivation and production and focused on work-related stresses. Therefore, non-work stresses and communication issues are excluded. In our study, we selected the appropriate dimensions and criteria for mental health factors, as shown in Table 1.

**Table 1:** Mental health factor model of work from home.

Dimension	Criteria
Work-related stressors (*C*_1_)	Workloads (*C*_11_)
	Work schedules (*C*_12_)
	Structural emptiness (*C*_13_)
	Unscheduled virtual meetings (*C*_14_)
	Weekend tasks (*C*_15_)
	High work expectations (*C*_16_)
	Job insecurity (*C*_17_)
Non-work stressors (*C*_2_)	Distraction (*C*_21_)
	Work–life imbalances (*C*_22_)
	Time management issues (*C*_23_)
	Domestic issues (or children’s presence at home) (*C*_24_)
	Laziness due to being at home (*C*_25_)
	An inconsistent sleep schedule (*C*_26_)
Communication issues (*C*_3_)	Lack of social interaction (*C*_31_)
	Improper guidelines (*C*_32_)
	No feedback exchange (*C*_33_)
	No proper collaboration with superiors and peers (*C*_34_)
	A lack of coordination (*C*_35_)

### The DEMATEL and DANP methods

The DEMATEL method is a system structure analysis that analyzes the complex social network structure of problems in the real world using the practical experience of a group of experts (22, 23). This method can analyze the interaction between subsystems in a system and visualize it using graph theory, to construct an influence network diagram (24–26). This diagram can help decision-makers focus on a few major influencing factors (27, 28). Subsequently, a novel method has been developed that transforms the influence weights from the total influence relation results of the DEMATEL method through the principle and characteristics of the ANP method. This method is also called DEMATEL-based ANP or DANP. This method has been applied to policy management (29), food safety risk management (30), and online shopping (24). The detailed calculation steps of this method are outlined in previous related research (31–33). The mathematical steps and calculation equations of this method are as follows:

Step 1: Based on the mental health factor model of working from home, all experts quantify the mutual influence among all factors (That is, the degree of influence of factor i on factor j , and we ask the same thing again in reverse), then the factors must pass a set of quantitative scale of influence relationships (i.e., 0: *no influence* to 4 *extremely high influence*). The experience matrix $E={\left[e_{ij}\right]}_{n\times n}$ of each expert can be constructed, and the matrix $A={\left[a_{ij}\right]}_{n\times n}$ representing respondents can be obtained by the averaging method. See Equation 1.


(1) $$A={\left[\begin{array}{ccccc} a_{11} & \cdots & a_{1j} & \cdots & a_{1n}\\ \vdots & \ddots & \vdots & \ddots & \vdots \\ a_{i1} & \cdots & a_{ij} & \cdots & a_{\mathrm{in}}\\ \vdots & \ddots & \vdots & \ddots & \vdots \\ a_{n1} & \cdots & a_{nj} & \cdots & a_{nn} \end{array}\right]}_{n\times n}={\left[\frac{\left(\sum_{\varepsilon =1}^{\vartheta }e_{ij}^{\varepsilon }\right)}{\vartheta }\right]}_{n\times n}$$

Step 2: Set the influence range boundary and convert the influence relationship degree to 0–1, as shown in Equations 2, 3.


(2) $$\delta =\max\left\{\max\overset{n}{\underset{j=1}{\sum }}a_{ij},\max\overset{n}{\underset{i=1}{\sum }}a_{ij}\right\}$$

(3) $$D=\frac{A}{\delta }$$

Step 3: Calculate the total degree of mutual influence between factors, and finally, produce the total influence relationship matrix T , as shown in Equation 4.


(4) $$T=D+D^{2}+\ldots +D^{\psi }=D{\left(I-D\right)}^{-1},\mathrm{when}\;\lim_{\psi \to \infty }D^{\psi }={\left[0\right]}_{n\times n}$$

Step 4: Derive the relevant analysis indexes of all factors, namely, “Give influence $u_{i}$ ,” “Receive influence $r_{i}$ ,” centrality, “Influence center, $u_{i}+r_{i}$ ” and “Influence cause or effect $u_{i}-r_{i}$ ,” as shown in Equations 5–8.


(5) $$u_{i}=\left(u_{1},u_{2},\ldots ,u_{n}\right)={\left[\overset{n}{\underset{j=1}{\sum }}t_{ij}\right]}_{n\times 1}$$

(6) $$r_{i}=\left(r_{1},r_{2},\ldots ,r_{n}\right)={\left(t_{j}\right)}_{1\times n}^{'}={\left[\overset{n}{\underset{i=1}{\sum }}t_{ij}\right]}_{1\times n}^{'}$$

(7) $$u_{i}+r_{i}$$

(8) $$u_{i}-r_{i}$$

“Give influence $u_{i}$ ” and “Receive influence $r_{i}$ ” represent the influence of factors and the affected values, respectively. When these two indices are added, they represent the influence intensity of the factor in the whole system, while subtracting them indicates the influence nature of this factor in the system, that is, cause or effect. The former is called “Influence center $u_{i}+r_{i}$ ,” and the latter is called “Influence cause or effect $u_{i}-r_{i}$ .”

Step 5: The boundary is established based on the total influence relation matrix T and converted into 0–1. The unweighted super matrix $\omega_{C}$ is derived, as shown in Equation 9.


(9) $$\begin{array}{l} \;\overset{D_{1}}{c_{11}\ldots c_{1m_{1}}}\cdots \;\overset{D_{o}}{c_{o1}\ldots c_{om_{o}}}\cdots \overset{D_{m}}{c_{m1}\ldots c_{mm_{m}}}\\ \omega_{C}=(T_{C}^{\alpha })'={\;}_{\begin{array}{l} D_{i}\\ \;\\ \vdots \\ \;\\ \;\\ D_{m} \end{array}}^{\begin{array}{l} D_{1}\\ \;\\ \vdots \\ \;\\ \; \end{array}}{\;}_{\begin{array}{l} \vdots \\ c_{im_{i}}\\ \;\\ \;\\ c_{m1}\\ \vdots \\ c_{mm_{m}} \end{array}}^{\begin{array}{l} c_{11}\\ \vdots \\ c_{1m_{1}}\\ \;\\ \;\\ c_{i1} \end{array}}{\left[\begin{array}{ccccc} T_{\alpha C}^{11} & \cdots & T_{\alpha C}^{1o} & \cdots & T_{\alpha C}^{1m}\\ \vdots & \; & \vdots & \; & \vdots \\ T_{\alpha C}^{i1} & \cdots & T_{\alpha C}^{io} & \cdots & T_{\alpha C}^{im}\\ \vdots & \; & \vdots & \; & \vdots \\ T_{\alpha C}^{m1} & \cdots & T_{\alpha C}^{mo} & \cdots & T_{\alpha C}^{mm} \end{array}\right]}_{n\times n\text{|}m<n,\;\sum_{o=1}^{m}m_{o}=n} \end{array}$$

Equation 10 shows the action of normalizing the total influence relation matrix (i.e., the values in the matrix are all between 0 and 1).


(10) $$\begin{array}{l} \;c_{11}\;\cdots \;c_{1o}\;\cdots \;c_{1m_{1}}\\ T_{\alpha C}^{11}={\;}_{\begin{array}{l} c_{1i}\\ \;\vdots \\ c_{1m_{1}} \end{array}}^{\begin{array}{l} c_{11}\\ \;\vdots \end{array}}\left[\begin{array}{ccccc} t_{C}{\;}_{11}^{11}/c_{1}^{11} & \cdots & t_{C}{\;}_{1o}^{11}/c_{1}^{11} & \cdots & t_{C}{\;}_{1m_{1}}^{11}/c_{1}^{11}\\ \vdots & & \vdots & & \vdots \\ t_{C}{\;}_{i1}^{11}/c_{i}^{11} & \cdots & t_{C}{\;}_{io}^{11}/c_{i}^{11} & \cdots & t_{C}{\;}_{im_{1}}^{11}/c_{i}^{11}\\ \vdots & & \vdots & & \vdots \\ t_{C}{\;}_{m_{1}1}^{11}/c_{m_{1}}^{11} & \cdots & t_{C}{\;}_{m_{1}j}^{11}/c_{m_{1}}^{11} & \cdots & t_{C}{\;}_{m_{1}m_{1}}^{11}/c_{m_{1}}^{11} \end{array}\right]\\ \;c_{11}\;\cdots \;c_{1o}\;\cdots \;c_{1m_{1}}\\ \;={\;}_{\begin{array}{l} c_{1i}\\ \;\vdots \\ c_{1m_{1}} \end{array}}^{\begin{array}{l} c_{11}\\ \;\vdots \end{array}}{\left[\begin{array}{ccccc} t_{\alpha C}{\;}_{11}^{11} & \cdots & t_{\alpha C}{\;}_{1o}^{11} & \cdots & t_{\alpha C}{\;}_{1m_{1}}^{11}\\ \vdots & & \vdots & & \vdots \\ t_{\alpha C}{\;}_{i1}^{11} & \cdots & t_{\alpha C}{\;}_{io}^{11} & \cdots & t_{\alpha C}{\;}_{im_{1}}^{11}\\ \vdots & & \vdots & & \vdots \\ t_{\alpha C}{\;}_{m_{1}1}^{11} & \cdots & t_{\alpha C}{\;}_{m_{1}o}^{11} & \cdots & t_{\alpha C}{\;}_{m_{1}m_{1}}^{11} \end{array}\right]}_{m_{1}\times m_{1}} \end{array}$$

Step 6: Transforming the unweighted super matrix into the weighted super matrix, i.e., the unweighted super matrix at the criterion level is adjusted by the conversion parameters at the dimension level, as shown in Equations 11, 12.


(11) $$\begin{array}{l} q^{D}=(T_{D}^{\alpha })'=\left[\begin{array}{ccccc} t_{11}^{D_{11}}/d_{1} & \cdots & t_{1o}^{D_{1o}}/d_{1} & \cdots & t_{1m}^{D_{1m}}/d_{1}\\ \vdots & & \vdots & & \vdots \\ t_{i1}^{D_{i1}}/d_{i} & \cdots & t_{io}^{D_{io}}/d_{i} & \cdots & t_{im}^{D_{im}}/d_{i}\\ \vdots & & \vdots & & \vdots \\ t_{m1}^{D_{m1}}/d_{m} & \cdots & t_{mj}^{D_{mo}}/d_{m} & \cdots & t_{mm}^{D_{mm}}/d_{m} \end{array}\right]\\ \;={\left[\begin{array}{ccccc} t_{11}^{\alpha_{11}} & \cdots & t_{1j}^{\alpha_{1o}} & \cdots & t_{1m}^{\alpha_{1m}}\\ \vdots & & \vdots & & \vdots \\ t_{i1}^{\alpha_{i1}} & \cdots & t_{io}^{\alpha_{io}} & \cdots & t_{im}^{\alpha_{im}}\\ \vdots & & \vdots & & \vdots \\ t_{m1}^{\alpha_{m1}} & \cdots & t_{mj}^{\alpha_{mo}} & \cdots & t_{mm}^{\alpha_{mm}} \end{array}\right]}_{m\times m} \end{array}$$

(12) $$\varpi =q^{D}\times \omega_{C}={\left[\begin{array}{ccccc} t_{11}^{\alpha_{11}}\times \omega_{C}^{11} & \cdots & t_{i1}^{\alpha_{i1}}\times \omega^{i1} & \cdots & t_{m1}^{\alpha_{m1}}\times \omega^{m1}\\ \vdots & & \vdots & & \vdots \\ t_{1o}^{\alpha_{i1}}\times \omega^{1o} & \cdots & t_{\mathrm{io}}^{\alpha_{\mathrm{io}}}\times \omega^{\mathrm{io}} & \cdots & t_{mo}^{\alpha_{mo}}\times \omega^{mo}\\ \vdots & & \vdots & & \vdots \\ t_{1m}^{\alpha_{m1}}\times \omega^{1n} & \cdots & t_{im}^{\alpha_{im}}\times \omega^{\mathrm{in}} & \cdots & t_{\mathrm{mm}}^{\alpha_{\mathrm{mm}}}\times \omega^{\mathrm{mm}} \end{array}\right]}_{n\times n|m<n,\;\sum_{o=1}^{m}m_{o}=n}$$

Step 6: The convergence process of the weighted super matrix through Markov chain calculation always reaches a steady state; that is, the influence weight of each factor is obtained, as shown in Equation 13.


(13) $$\varpi^{*}=\lim_{\rho \to \infty }{\left(\varpi \right)}^{\rho }$$

### Ethics approval

This study was approved by the Ethics Committee of Taizhou Central Hospital (Taizhou University Hospital) (Grant No. 2023L-05-07), it was conducted following the ethical guidelines described in the Declaration of Helsinki. The purpose was explained in detail to the experts before the investigation, and their consent was obtained during the investigation. Participants could terminate or withdraw from the study at any time during the study period.

### Data collection and participants

The questionnaire is based on the characteristics of the DEMATEL method. At the same time, to increase the validity and reliability of data collection, the investigators adopted the purposive survey method and explained the purpose and significance of this study in person. Respondents agreed to participate in the study and fill out the questionnaire. Mental health survey data were collected from 33 hospital administrators who worked from home during the COVID-19 epidemic. The consensus gap of experts on this data result is 0.0121. In other words, the confidence level reaches 98.79% (i.e., the consensus gap is 1.12%). The survey was conducted from May to June 2023.

## Results

### Data presentation

In this questionnaire survey, there was little difference between men and women (55% men and 45% women), and their ages were mainly over 30 years (*n* = 28, 85%); most had a university education (*n* = 21, 64%), and most had worked for 10 years or more (*n* = 20, 61%). Furthermore, all respondents had practical experience of working from home and 58% worked from home for 2 weeks or more during the epidemic. The backgrounds of all respondents are shown in Table 2.

**Table 2:** Demographic characteristics of 33 hospital administrators.

Characteristics	Value (%)
Sex	
Male	18 (55%)
Female	15 (45%)
Age	
<30	5 (15%)
30–39	19 (58%)
≥40	9 (27%)
Education	
Bachelor	21 (64%)
Master or above	12 (36%)
Years of service	
Under 10 years	13 (39%)
10–15	9 (27%)
15 and above	11 (34%)
Professional title	
Technologist-in-charge	3 (9%)
Supervisor nurse	3 (9%)
Senior technologist	2 (6%)
Senior nurse	5 (15%)
Senior engineer	1 (3%)
Senior doctor	1 (3%)
Senior accountant	2 (6%)
Researcher	1 (3%)
Registered nurse	2 (6%)
Librarian	1 (3%)
Engineer	1 (3%)
Economic engineer	1 (3%)
Doctor	3 (9%)
Chief nurse	1 (3%)
Associate professor	1 (3%)
Associate chief physician	2 (6%)
Accountant	3 (9%)
Experience in working from home	
Yes	33 (100%)
No	0 (0%)
Working hours from home	
<2 weeks	14 (42%)
2–3 weeks	10 (30%)
3–4 weeks	4 (12%)
≥4 weeks	5 (16%)

### Network relation map

The relationship between psychological factors of working at home in 33 respondents during the COVID-19 epidemic can be analyzed by “Influence center $u_{i}+r_{i}$ ” and “Influence cause or effect $u_{i}-r_{i}$ .”

From the perspective of the “Influence center $u_{i}+r_{i}$ ,” “Non-work stressors” (*C*_2_) is the center of gravity for all mental health factors, and it has the highest interplay correlation compared to the other two dimension levels. Additionally, “Workloads” (*C*_11_), “Work schedules” (*C*_12_), and “Distraction” (*C*_21_) were clearly the top three highest correlations of interactions with all factors compared to other mental health factors.

**Figure 2:**
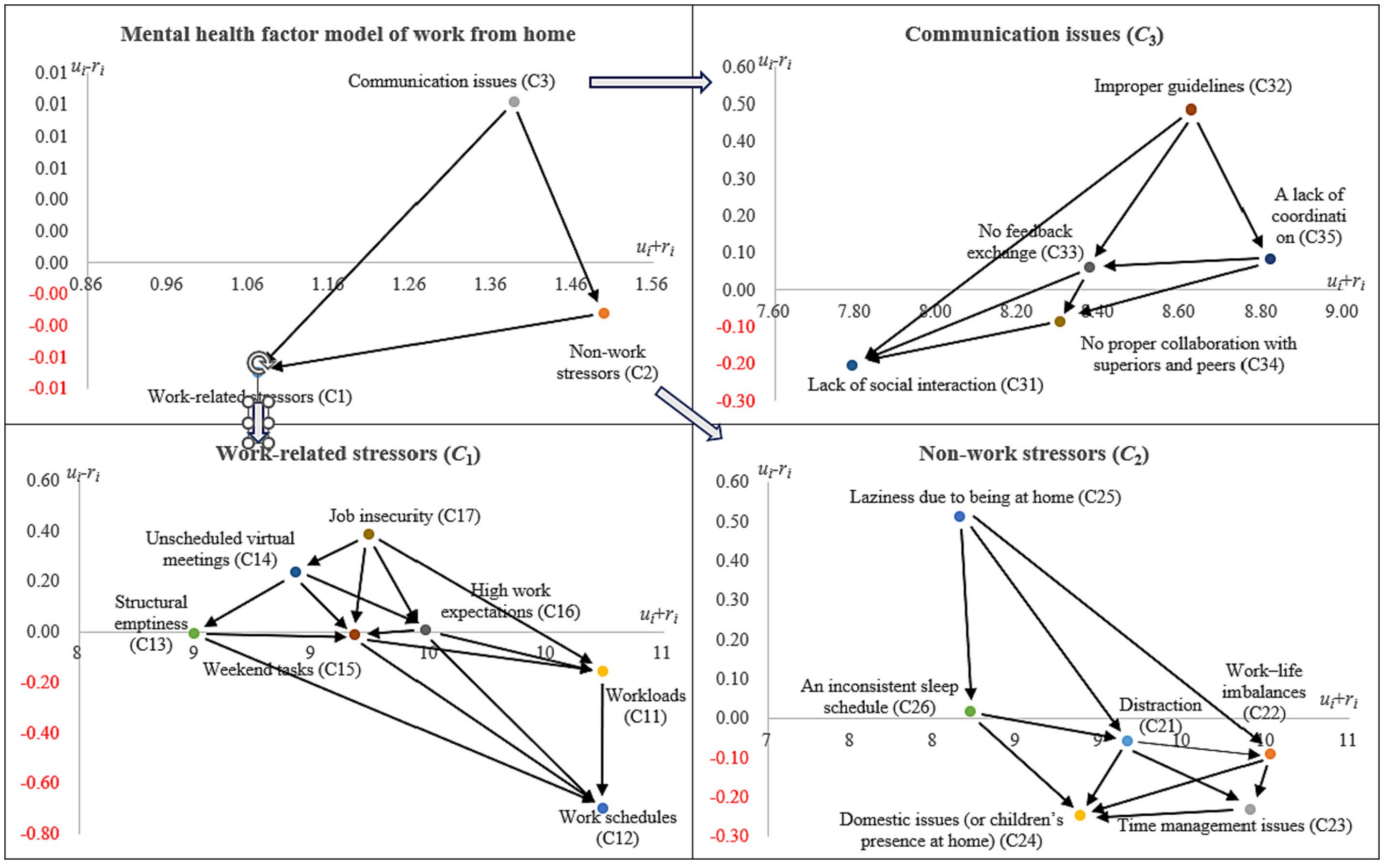
The influential network relation map (INRM).

From the perspective of the “Influence cause or effect $u_{i}-r_{i}$ ,” in the dimension level, the “Work-related stressors” (*C*_1_) and “Non-work stressors” (*C*_2_) are the effect groups; “Communication issues” (*C*_3_) is the influence group. However, in the criteria level, “Workloads” (*C*_11_), “Work schedules” (*C*_12_), “Structural emptiness” (*C*_13_), “Weekend tasks” (*C*_15_), “Distraction” (*C*_21_), “Work-life imbalance” (*C*_22_), “Time management issues” (*C*_23_), “Domestic issues (or children’s presence at home)” (*C*_24_), “Lack of social interaction” (*C*_31_), “No proper collaboration with superiors and peers” (*C*_34_)” are the effect group; “Unscheduled virtual meetings” (*C*_14_), “High work expectations” (*C*_16_), “Laziness due to being at home” (*C*_25_), “An inconsistent sleep schedule” (*C*_26_), “Improper guidelines” (*C*_32_), “No feedback exchange” (*C*_33_), and “A lack of coordination” (*C*_35_) are the cause group. Table 3 shows the results of the impacts of all factors and further shows the structure of the interrelationships between all factors by means of Figure 2, i.e., the influential network relation map (INRM).

**Table 3:** The influential network structure of mental health factors.

Factors	Give influence	Receive influence	Influence center	Influence cause and effect
C_1_	0.532	0.539	1.070	−0.007
C_11_	5.043	5.197	10.240	−0.155
C_12_	4.770	5.468	10.238	−0.699
C_13_	4.241	4.248	8.489	−0.007
C_14_	4.581	4.345	8.925	0.236
C_15_	4.584	4.596	9.180	−0.013
C_16_	4.745	4.735	9.480	0.010
C_17_	4.813	4.427	9.240	0.386
C_2_	0.748	0.751	1.498	−0.003
C_21_	4.553	4.611	9.164	−0.057
C_22_	4.965	5.056	10.021	−0.091
C_23_	4.834	5.064	9.897	−0.230
C_24_	4.316	4.564	8.880	−0.248
C_25_	4.334	3.821	8.156	0.513
C_26_	4.118	4.102	8.220	0.016
C_3_	0.699	0.689	1.388	0.010
C_31_	3.794	3.997	7.791	−0.202
C_32_	4.555	4.070	8.625	0.484
C_33_	4.218	4.158	8.375	0.060
C_34_	4.109	4.195	8.304	−0.085
C_35_	4.451	4.367	8.818	0.083

### Influence weight analysis

The influence weight represents the degree of influence of a factor on mental health in work from home. The higher the value, the more attention should be paid to this factor. For the dimensions, “Non-work stressors” (*C*_2_) has the highest influence weight, followed by “Communication issues” (*C*_3_) and “Work-related stressors” (*C*_1_), from the local perspective. Furthermore, the highest weights in each dimension are “Time management issues” (C_23_), “A lack of coordination” (C_35_), and “Work schedules” (C_12_), which represent the local weight perspective. Finally, “A lack of coordination” (C_35_), “Time management issues” (C_23_), and “Work-life imbalance” (C_22_) are the top three criteria in terms of the global perspective. The influence weight results for all the factors are listed in Table 4.

**Table 4:** The influential weights of mental health factors.

Factors	Local weight	Rank	Global weight	Rank
C_1_	0.271	3		
C_11_	0.158	2	0.043	13
C_12_	0.166	1	0.045	12
C_13_	0.129	7	0.035	18
C_14_	0.131	6	0.036	17
C_15_	0.139	4	0.038	15
C_16_	0.143	3	0.039	14
C_17_	0.135	5	0.037	16
C_2_	0.380	1		
C_21_	0.169	3	0.064	8
C_22_	0.186	2	0.071	3
C_23_	0.186	1	0.071	2
C_24_	0.168	4	0.064	9
C_25_	0.141	6	0.053	11
C_26_	0.151	5	0.057	10
C_3_	0.349	2		
C_31_	0.192	5	0.067	7
C_32_	0.196	4	0.068	6
C_33_	0.200	3	0.070	5
C_34_	0.202	2	0.070	4
C_35_	0.210	1	0.073	1

## Discussion

### Interpretation of findings based on the influence weight

In this study, “A lack of coordination” (C_35_), “Time management issues” (C_23_), and “Work-life imbalance” (C_22_) are the key influencing factors. In the “Communication issues” (C_3_) dimension, “A lack of coordination” (C_35_) is a key influencing factor. A study of four Japanese manufacturing companies found differences in intra-company productivity between those who worked from home and those who did not during the COVID-19 pandemic. The results show that poor remote working environments, communication, and coordination are the main reasons for the decline in productivity (34). Poor communication in the workplace and with customers had significant negative effects. Face-to-face communication can effectively reduce the negative effects of uncoordinated communication. Amano et al. (35) found that during the COVID-19 pandemic, close communication between employees working at home and top leaders was a key factor affecting employee engagement. Another systematic review found that most studies showed that people who transition to work from home for the first time are most likely to be less productive than normal (36). Furthermore, one study showed that administrative staff who work remotely are worried about the lack of organizational communication and teamwork, which will affect their current work (37). In summary, a lack of communication and coordination may lead to mental health problems while working from home and may even aggravate the generation of negative emotions, resulting in a further reduction in productivity.

In the “Non-work stressors” (C_2_) dimension, “Time management issues” (C_23_) is an influencing factor. One study showed (38) that during periods when employees perform some or all of their job responsibilities at home, the time spent on childcare, housework, family dining, and preparation also increases significantly. Furthermore, the study found that even on the premise that the processing time of family affairs increased greatly, the time spent in remote work also showed an increasing trend. Therefore, people who work remotely from home experience great pressure on time management and need to pay more attention to it. Hospital administrators working remotely experience negative mental health effects.

In the “Non-work stressors” (C_2_) dimension, “Work-life imbalance” (C_22_) is another key influencing factor. In a literature review on the impact of COVID-19 on telecommuting employees, employees who were forced to switch to telecommuting because of the pandemic faced work–family conflicts and work overload, which can generate greater stress, accelerate fatigue, and reduce telecommuting satisfaction and job performance (36). In addition, Chu, Chan (39) showed that it is very important for management to maintain a healthy work-life balance for employees who work from home to support their mental health and improve their work efficiency. Among the three stress relief methods studied, work-life balance is the only one that affects employees’ mental health. Therefore, during remote work, hospital administrators experience conflicts between work and family, which may lead to negative emotions and affect their mental health.

### Implications based on the INRM

“Influence center” and “Influence cause or effect” can show the structure of the network relationship between all factors, namely the INRM, as shown in Figure 2. The Figure shows that “Improper guidelines” (C_32_), “Laziness due to being at home” (C_25_), and “Job insecurity” (C_17_) are the most important factors needing improvement in all dimensions. Therefore, hospital decision-makers can propose improvement schemes from the above three factors and combine them with their practical experience in hospital management.

During the COVID-19 pandemic, telecommuting is no longer a unique working mode but has become an effective supplement to the traditional working mode. Hospital administrators’ main tasks include writing documents, data analysis, and communication. Incorrect guidelines will affect the performance of remote work, which will lead to poor work outcomes and indirectly cause psychological pressure.

This study concludes that addressing “Improper guidelines” (C_32_) is an effective improvement factor, and improvement measures can be proposed from two levels of hospital managers and hospital administrative staff. At the management level, hospital leaders and department managers need to acknowledge that telecommuting has become an indispensable part of their daily work, and the advantages and disadvantages of telecommuting, including productivity, job performance, and mental health, must be fully considered.

The guidelines issued by leaders have a significant influence on the aforementioned telecommuting problems of managers and cannot provide work guidance according to the traditional working mode. Therefore, the guidelines for remote work at the leadership level, including work content, completion, quality requirements, and data collection, should be clear. Managers need to actively communicate fully with the administrative staff who work remotely, listen to the opinions of the work implementers, and adjust the contents of the guidelines to ensure they are practical and can be implemented remotely.

In addition, management styles can lead to inappropriate guidelines. Such leaders often have high authority, do not allow others to express their opinions about work, and require attention. When hospital administrators receive remote work instructions from managers, they should not blindly implement them and instead immediately confirm the work content, completion time, and work requirements with directly affiliated managers. Before starting work, the feasibility of the remote working mode should be analyzed, and opinions for managers’ reference should be put forward to avoid improper guidance affecting remote work.

This study found that addressing “(C_25_)” laziness caused by being at home is another effective improvement factor. When administrators work in hospitals, the working environment includes constraints and supervision factors such as working hours and peer supervision, which can ensure work efficiency. When working remotely, the restrictions and supervision factors of the hospital working environment disappear, and laziness occurs at home, leading to lower work efficiency and longer working hours. Improvement strategies can be proposed based on these five perspectives.

In terms of personal ability, hospital administrators can improve their self-discipline and time management ability. Self-discipline is the primary factor that affects personal work efficiency. Some studies have shown that employees who think they are self-disciplined are more active, effective, and timely in time management than those who think they are not (36). Therefore, improving the quality of self-discipline and time management ability of hospital administrators can improve the efficiency of remote work and reduce negative emotions such as anxiety and uneasiness caused by laziness and lack of self-discipline.

At the hospital organization system level, hospitals should supervise the remote work of administrators and adopt flexible working hours. Supervision is an important management strategy for overcoming laziness. Hospitals can supervise the effects of telecommuting through regular work reports and inspections, urge telecommuting managers to begin work on time, avoid unreasonable time arrangements caused by laziness, objectively reduce the probability of laziness at home, and reduce the negative emotions and psychological states caused by laziness. Flexible working hours should be adopted. Anyone working remotely has their own unique and efficient working hours. By adopting a flexible working-hours mode, teleworkers can use their working hours efficiently to complete their work and objectively reduce their laziness.

At the hospital logistics support and humanistic care levels, hospital administrators should control fatigue when working remotely. Some studies have shown that owing to factors such as home place, office conditions, and working hours, the probability of muscle soreness and eye fatigue in home telecommuting is higher than that in office places (38). From another perspective, fatigue and discomfort lead to increased fatigue in telecommuters, and some administrators may increase the likelihood of laziness. Therefore, hospitals should provide hardware support for telecommuters, such as ergonomic office chairs and proper lighting, which can effectively reduce fatigue while working from home. At the psychological level, such people can also feel the support of the organization, improve their motivation within the work, and reduce the possibility of laziness.

This study also found that addressing “Job insecurity” (C_17_) and job instability are also effective improvement factors. Hospital administrators generally have clear job responsibilities and work plans, and telecommuting leads to significant changes in their work content, workload, and working hours. These aspects are unstable, and hospital administrators are prone to psychological pressures, such as anxiety and irritability. Therefore, improvement strategies are proposed for these three levels.

Regarding the work content level, additional tasks may be added during remote work, including newly added temporary work, to enable collaboration with colleagues to complete the work. During the COVID-19 pandemic, hospital administrators added considerable temporary work to data reporting and documentation and needed to provide work assistance to colleagues who were resting. The above work contents are all new tasks, and administrators who need to work remotely are particularly unfamiliar with the new process and work content, which causes tension and anxiety.

When managers arrange new tasks, they should plan and decompose the work content and arrange people with similar work content or relevant skills. In addition, we should pay attention to the problems encountered in the process of carrying out new work and help solve them promptly. Facing new work tasks, managers who work remotely adjust their psychological state over time, make work plans and support conditions, and report to them to obtain work guidance and support (39).

Regarding workload and working hours, this depends mainly on the task itself and organizational factors. Each task has its own work content and time-limit requirements, which directly determine the workload and working hours. Managers should consider the sum of the workload and working hours of each executive and try their best to achieve balance.

Organizational factors include organizational design and leadership style. For example, during the COVID-19 pandemic, the medical management department undertook most of the prevention and control management and data statistics, as well as much coordination work. The new workload and working hours increased significantly, but compared with other administrative departments, there was no obvious increase. In view of this phenomenon, breaking the traditional bureaucratic structure and implementing the project structure in some posts can effectively adjust the workload and working hour pressure of key departments.

In addition, a positive leadership style can elicit positive emotions from team members, making employees feel that their organization is taking care of them and that their work can develop positive emotional resources. In these cases, current and caring leadership styles represent an appropriate form of organizational support that can effectively reduce the psychological stress caused by bad emotions.

## Limitations

This study had several limitations. First, the participants in this study were recruited through a purposeful sampling method, which may have led to sampling deviation. In addition, the results were limited to the investigation of the case hospital at that time and should not be inferred from subsequent time points or other hospitals. Finally, the method used in this study aimed to obtain the influence network structure and corresponding weights from the perspective of influence, which is different from the preference relationship weighting method (such as the analytic hierarchy process).

## Conclusion

Based on the weights of this study and the main results of INRM found that “A lack of coordination” (C_35_), “Time management issues” (C_23_), and “Work-life imbalance” (C_22_) are the main factors affecting mental health; and that “Improper guidelines” (C_32_), “Laziness due to being at home” (C_25_), and “Job insecurity” (C_17_) are the most important factors that need to be improved across all dimensions. Therefore, hospital decision-makers can start from the above three factors and combine them with the actual experience of hospital management to propose improvement programs. Also, scholars can further study the mental health factors of home-based workers from different perspectives, including different roles (i.e., teachers), different health factors (i.e., adding other factors), and analyzing different decision-making methods (i.e., from different decision-making perspectives). These are all future research directions that will help hospital decision-makers take early preventive measures for home office mental health problems in the face of potential major infectious diseases in the future.

## Data availability statement

The raw data supporting the conclusions of this article will be made available by the authors, without undue reservation.

## Ethics statement

The studies involving humans were approved by the Ethics Committee of Taizhou Central Hospital (Taizhou University Hospital) (Grant no. 2023L-05-17) and was conducted in accordance with the ethical guidelines described in the Declaration of Helsinki. The purpose was explained in detail to the experts before the investigation, and their consent was obtained during the investigation. Participants could terminate or withdraw from the study at any time during the study. The studies were conducted in accordance with the local legislation and institutional requirements. The participants provided their written informed consent to participate in this study.

## Author contributions

SS: Writing – original draft, Investigation. JZ: Writing – original draft, Formal analysis. WS: Data curation, Methodology, Writing – original draft. Y-CC: Methodology, Supervision, Writing – original draft, Writing – review & editing. CL: Formal analysis, Validation, Writing – original draft. HL: Project administration, Supervision, Writing – review & editing.
